# *Neurospora crassa* mat A-2 and mat A-3 proteins weakly interact in the yeast two-hybrid system and affect yeast growth

**DOI:** 10.1590/S1415-47572009000200023

**Published:** 2009-06-01

**Authors:** Carla C. da Silva, Rosana C. Cruz, Mônica Bucciarelli-Rodriguez, Adlane Vilas-Boas

**Affiliations:** Departamento de Biologia Geral, Instituto de Ciências Biológicas, Universidade Federal de Minas Gerais, Belo Horizonte, MGBrazil

**Keywords:** * Neurospora crassa*, mating-type, yeast two-hybrid system

## Abstract

*Mating-type* genes control the entry into the sexual cycle, mating identity and sexual development in fungi. The *mat A-2* and *mat A-3* genes, present in the *mat A* idiomorph of the filamentous fungus *Neurospora crassa*, are required for post-fertilization functions but are not essential for mating identity. Their putative roles as transcription factors are based on the similarity of *mat A-2* with the *Podospora anserina SMR1* gene and an HMG motif present in the *mat A-3* gene. In this work the yeast two-hybrid system was used to identify transcriptional activity and protein-protein interaction of *N. crassamat A-2* and *mat A-3* genes. We observed that the mat A-3 protein alone is capable of weakly activating transcription of yeast reporter genes; it also binds with low specificity to the *GAL1* promoter sequence, possibly due to its HMG domain. Our results also indicate that mat A-3 is capable to form homodimers, and interact with mat A-2. Interference on yeast growth was observed on some transformants suggesting a toxic action of the mat A-2 protein. Our data on pattern of interactions of mat proteins contributes towards understanding the control of vegetative and sexual cycles in filamentous fungi.

## Introduction

Entry into the sexual cycle in fungi is controlled by the *mating-type (mat)* genes which are responsible for sexual development and identity in filamentous fungi and yeast (reviewed in Kronstad and Staben, 1997; Souza *et al.*, 2003). Although it is known that many *mat* gene products are transcription factors, very little is known about how MAT proteins interact and what are the target genes in filamentous ascomycete fungi.

*N. crassa* was the first filamentous fungus that had its *mat* sequences cloned and physically characterized (Glass *et al.*, 1988). The *mat A* and *mat a* are non-homologous sequences present at the *mat* locus, thus called idiomorphs (Metzenberg and Glass, 1990). The *mat a* idiomorph contains the *mat a-1* gene which is responsible for sexual identity, vegetative incompatibility and perithecium development (Staben and Yanofsky, 1990). The *mat a-1* gene has a DNA-binding HMG motif, found in certain transcriptional regulatory proteins, and was shown to regulate the *mfa-1* pheromone precursor gene among other genes (Kim *et al.*, 2002). A second ORF, *mat a-2,* is also present in the *mat a* idiomorph, but is apparently not transcribed (Pöggeler and Kuck, 2000). The *mat A* idiomorph contains three genes: *mat A-1*, *mat A-2* and *mat A-3* (Glass *et al.*, 1990; Ferreira *et al.*, 1996). The *mat A-1* gene product contains an α1-domain, similar to *Saccharomyces cerevisiae* MATα1 protein. The mat A-1 protein is sufficient and required for vegetative incompatibility, sexual identity, sexual development and production of A-specific pheromones and a-pheromone receptors (Glass *et al.*, 1990; Bobrowicz *et al.*, 2002). Ferreira *et al.* (1996, 1998) have characterized the additional two *N. crassa mat* genes: *mat A-2* codes for a novel putative DNA-binding domain protein, and *mat A-3*, as the *mat a-1* gene, codes for an HMG domain protein. The *mat A-2* and *mat A-3* genes are similar to the *SMR1* and *SMR2 mat* genes of *P. anserina,* respectively (Ferreira *et al.*, 1996), which are necessary for post-fertilization events in this fungus (Debuchy *et al.*, 1993). Despite the similarity, *N. crassa mat* genes are not capable of complementing *P. anserinaSMR1* and *SMR2* mutants for post-fertilization events (Arnaise *et al.*, 1993). Also, phenotypic analysis of *mat A-2* and *mat A-3* mutants indicated that these genes function differently from their homologues (Ferreira *et al.*, 1998), since a mutant phenotype is only observed when both genes are mutated. A question that arises from this intriguing observation is whether mat A-2 and mat A-3 act independently or an interaction between them is needed for their function.

Protein-protein interaction in the yeast two-hybrid (Y2H) system has been observed between *N. crassa* mat A-1 and mat a-1 proteins (Badgett and Staben, 1999) and between *P. anserina* FMR1 and SMR2 proteins (Debuchy and Coppin, unpublished data cited in Coppin *et al.*, 1997). The four *mat* genes of the homothallic pyrenomycete *Sordaria macrospora* (*Smta-1, SmtA-1, SmtA-2* and *SmtA-3*) are all highly similar to *N. crassamat* genes (Pöggeler *et al.*, 1997). The yeast two-hybrid system was also used to demonstrate that Smta-1 and SmtA-1 proteins interact and that these proteins contain domains capable of activating transcription in yeast (Jacobsen *et al.*, 2002). However, molecular interaction data on the *N. crassamat A-2* and *mat A-3* gene products or their homologues are not available. In this work we used the Y2H system to verify possible protein-protein interactions and transcriptional activation activity of the *N. crassamat A-2* and *mat A-3* gene products.

**Figure 1:**
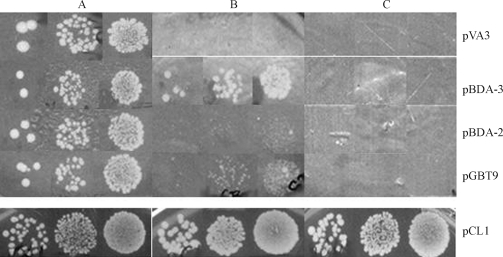
Transcriptional activation in 3-AT assay. HF7c transformant cultures carrying plasmids pVA3 (pGBT9-*p53*), pBDA-3 (*GAL4-BD-mat A-3*), pBDA-2 (*GAL4-BD-mat A-2*), pGBT9 (vector) or pCL1 (entire *GAL4*), were spotted, at 10^-4^, 10^-3^ and 10^-2^ dilutions of a stationary culture, on SD medium lacking: A) tryptophan (for pVA3, pBDA-3, pBDA-2 and pGBT9) or leucine (for pCL1), B) tryptophan and histidine or leucine and histidine and C) tryptophan and histidine or leucine and histidine and both enriched with 2.5 mM 3-AT.

**Figure 2:**
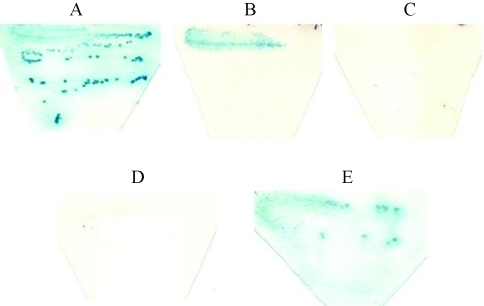
Transcriptional activation in the X-gal filter assay. Y190 transformant cultures carrying plasmids pCL1 (**A**; strong transcriptional activator), pNTmKad4 (**B**; weak transcriptional activator), pGBT9 (**C**; vector), pBDA-2 (**D**; GAL4-BD-mat A-2) or pBDA-3 (**E**; GAL4-BD-mat A-3) were grown on filters laid on solid SD medium lacking tryptophan. Filters were incubated at 37 °C for 4-16 h.

**Figure 3:**
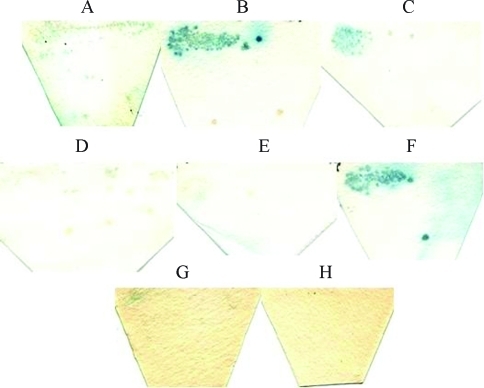
Two-hybrid X-gal filter assay. Transformant Y190/pGAD424 (**A**; original plasmid) was used as negative control while transformant Y190/pVA3/pTD1 (**B**; *GAL4-BD-p53*/*GAL4-AD-sv40 large T antigen*) was used as positive control for protein-protein interaction. Y190/pADA-3 (**C**; *GAL4-AD-matA-3*) and Y190/pADA-2 (**D**; *GAL4-AD-mat A-2*) were used as negative controls for transcriptional activation. These transformants were grown on SD media lacking leucine while Y190/pVA3/pTD1, Y190/pBDA-2 (*GAL4-BD-matA-2*)/pADA-2 (**E**), Y190/pBDA-3 (*GAL4-BD-mat A-3*)/pADA-2 (**F**), Y190/pBDA-3/pADA-3 (**G**) and Y190/ pBDA-2/pADA-3 (**H**) were grown on SD media lacking both tryptophan and leucine.

**Figure 4:**
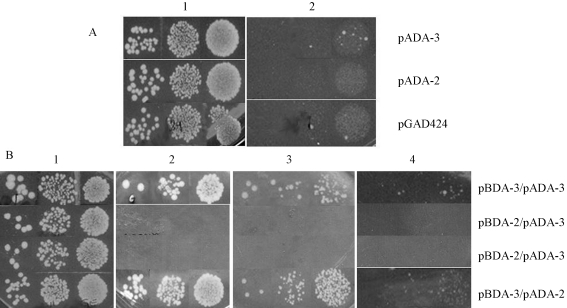
Two-hybrid 3-AT assay. Drops of HF7c transformant cultures carrying plasmids pADA-3 (*GAL4-AD-mat A-3*), pADA-2 (*GAL4-AD-mat A-2*), pGAD424 (vector) **(A)**, and combinations pBDA-3 (*GAL4-BD-mat A-3*)/pADA-3, pBDA-2 (*GAL4-BD-mat A-2*)/pADA-3, pBDA-2/pADA-2 and pBDA-3/pADA-2 **(B)** were applied on SD solid media. **A**) SD media devoid of leucine (**1**), for control purpose, and devoid of leucine and histidine (**2**). **B**) SD media devoid of leucine and tryptophan (control) (**1**); leucine, tryptophan and histidine (**2**); leucine, tryptophan and histidine enriched with 2.5 mM 3-AT (**3**) and 5 mM 3-AT (**4**).

## Materials and Methods

### Strains and media

*Saccharomyces cerevisiae* HF7c (*Mat a, ura3-52, his3-200, lys2-801, ade2-101, trp1-901, leu2-3/112, gal4-542, gal80-538, LYS2::GAL1-HIS3, URA3:*(*GAL4 17-mer*_*3*_)*-CYC-lacZ*) and Y190 (*MAT*a *trp1-901 his3 leu2-3,112 ura3-52 ade2 gal4 gal80 URA3*::*GAL1-lacZLYS2*::*GAL1-HIS3*) strains (Clontech Laboratories, Inc.) were used for the two-hybrid experiments. Isogenic IH 1783 (*MAT* a *trp1 leu2 ura3 his4 can1)*, IH1784 (*MAT* α *trp1 leu2 ura3 his4 can1)* and diploid IH1788 strains (*MAT* a/α *trp1 leu2 ura3 his4 can1*) (Michaelis and Herskowitz, 1988) were also used. YPD medium was used for propagation and SD medium, with appropriate nutrients, for selection of transformants. Recombinant plasmids were cloned and propagated in *Escherichia coli* MC1061 strain (Clontech Laboratories, Inc.) using standard conditions (Sambrook *et al.*, 1989).

### Construction of recombinant plasmids

Primers for PCR amplification of *mat A-2* and *mat A-3* genes were designed using the *mat A* idiomorph sequence (GenBank M33876); restriction enzyme sites were included at their 5' ends to facilitate cloning (Table 1). Amplification reactions performed in a PT-100 thermal cycler (MJ Research, Inc.) were set up in a final volume of 20 μL using primers (Imprint do Brazil Ltda, Campinas, Brazil) and one unit of *Taq* polymerase (Phoneutria Biotecnologia & Serviços, Belo Horizonte, Brazil) in a standard protocol. Plasmid pRAUW84 (Aramayo and Metzenberg, 1996), which has the *Nsi* I/*Nde* I *mat A* idiomorph fragment containing all three *mat A* genes, was used as template.

Plasmids pGBT9, pGAD424, pVA3, pCL1 and pTD1 were obtained from Clontech Laboratories, Inc.. The pGBT9, pGAD424 plasmids were used for cloning the PCR fragments for the Y2H experiments. pGBT9 contains the GAL4 DNA-binding domain (BD) sequence and the *TRP1* gene as selectable marker, while pGAD424 contains the GAL4 activation domain (AD) sequence and the *LEU2* gene as selectable marker. For the cloning of the *mat A-3* amplicon, both plasmids were restriction digested and treated with calf intestinal alkaline phosphatase (Promega Corp., Madison, WI, USA) after digestion for dephosphorylation of 5' overhangs. *N. crassa mat A-2* and *mat A-3* amplicons were purified and digested with the appropriate enzymes for in phase cloning into pGBT9 and pGAD424. Correct cloning was confirmed by PCR using BONO and EDGE primer pairs and restriction analysis.

### Two-hybrid experiments

Yeast cells were transformed following the protocol of Schiestl and Gietz (1989). Transformants were selected by screening for tryptophan, leucine or tryptophan/leucine prototrophy and transferred to SD with necessary requirements. To reduce the number of false positives in the yeast assays, the *LacZ* and the *HIS3* reporter genes were tested. The X-gal filter assay, to detect *LacZ* expression, was performed based on the protocol described in the MATCHMAKER Random Peptide Library User Manual PT3039-1 (Clontech Laboratories, Inc.) with modifications. Y190 transformants were grown on sterilized filters on solid SD media without tryptophan, leucine or neither amino acids for three days. Filters with yeast colonies were transferred to clean, sterilized plates and yeast cells were lysed using liquid nitrogen followed by thawing at room temperature. When filters were wet, a solution containing 2.81 mL of Z-buffer (60 mM Na_2_HPO_4_, 40 mM NaH_2_PO_4_, 10 mM KCl, 1 mM MgSO_4_), 7.6 μL of β-mercaptoethanol and 26.4 μL of X-gal (20 mg/mL) per plate was poured over the filters and the plates were incubated at 37 °C for 4-16 h. Cleavage of X-gal leaves a blue color on and around the yeast colonies, being a good indicator of *LacZ* gene expression. For the HIS3 reporter assay, HF7c transformants were grown on solid SD media without tryptophan, leucine, or both amino acids, and without histidine. An inhibitor of the enzyme encoded by the *HIS3* gene, 3-amino-1,2,4-triazole (3-AT), was used at 2.5 mM, 5 mM, 10 mM and 25 mM. This technique (which from now on we will call “3-AT assay”) allows observing whether there was activation of the *HIS3* reporter gene and helps to determine the strength of the activation. Cultures were diluted at 10^-2^, 10^-3^ and 10^-4^ and spotted on plates. Plates were incubated at 30 °C for 5-7 days.

Y190 and HF7c cells transformed with plasmids pGBT9 and pGAD424, respectively, were used as negative control of activation. Plasmid pVA3, containing the *p53* gene lacking its transcriptional activation domain fused to the GAL4p-BD, was used as the control for no transcription activation. Other control transformants bore: plasmid pCL1 which contains the whole *S. cerevisiae GAL4* gene and is thus a strong activator; and pNTMKad4 (Rodrigues LB, MSc Thesis, Universidade Federal de Minas Gerais, Belo Horizonte, MG, Brazil, 2001), containing a mutated form of the mTEAD1 transcriptional activator, a weak activator. pTD1 has the *SV40 large T antigen* gene fused to GAL4p-AD and was used in combination with pVA3 as the positive control for protein-protein interaction in the X-gal assay. Plasmid pCL1 was used as positive control for the HF7c strain in the mono-hybrid experiments.

### Growth curves

The different Y190 transformants were grown overnight at 30 °C in an orbital shaker in 5 mL of selective liquid SD media. They were then re-inoculated in 5 mL of the same medium at 1 x 10^6^ cells/mL and grown for two hours in a shaker (150 rpm) at 30 °C. At first, the same growth conditions were used for HF7c transformants; but, due to the extended lag phase of the latter (see results), the re-inoculation step was modified as follows: HF7c transformants were grown overnight at 30 °C in an orbital shaker (150 rpm) and cells were counted. HF7c/pGBT9 and HF7c/pBDA-3 transformants were inoculated at 0.5 x 10^4^ cells/mL. HF7c/pBDA-2 and HF7c/pBDA-2/ pADA-3 transformants were inoculated at 0.5 x 10^5^ cells/mL. All inocula were set up in 5 mL of selective liquid media lacking selected amino acids. Cultures were grown at 30 °C under 150 rpm for 16 h. Cells were counted at intervals of two hours in a hemocytometer until obtaining four points in the exponential growth curve. Each growth curve was repeated at least three times. The first point of the exponential growth curve was considered as time zero. The mean doubling time of the transformants was calculated using the inclination of the hypothetical exponential growth curves. Graphs were drawn using the Origin 7.5 software (OriginLab Corporation). Statistical analyses were performed using Student's *t*-test at p < 0.05 using the Statistica program.

### Plasmid cure experiments

pGBT9, pBDA2, pBDA3 and pBDA2/pADA3 transformants were grown on YPD for 48 h. Then, cells were counted and diluted to attain 300 CFU in an aliquot of 100 μL of each culture that were spread over YPD and selective SD plates. SD medium lacked histidine, leucine and adenine. Plates were incubated at 30 °C and colonies were counted after 72 h. Relative survival was calculated and shown as percentage.

## Results and Discussion

Potential transcriptional activation or silencing of a gene can be detected in yeast reporter gene systems when they are fused to the *GAL4DNA-binding* domain (Fields and Song, 1989). *N. crassa mat A-2* and *mat A-3* gene products are thought to be transcription factors (Ferreira *et al.*, 1996) and since their description, biochemical data such as those from DNA binding assays have not been reported. Thus to investigate the biochemical functions of the mat A-2 and mat A-3 proteins we decided to use the yeast reporter system. Both *mat A-2* and *mat A-3* sequences were cloned into plasmid pGBT9 fused to the *S. cerevisiae* GAL4p DNA-binding domain.

Transformants carrying pBDA-2 (*GAL4-BD-mat A-2*) failed to show reporter gene expression in the two systems, as shown by the lack of growth in medium without histidine and no production of blue colonies (Figures 1B, 2D and respectively). This indicates that *mat A-2* does not act as an activator of the systems by itself. On the other hand, pBDA-3 (*GAL4-BD-mat A-3*) transformants exhibited some transcriptional activation of both reporter genes (Figures 1B and 2E). The results suggest a weak activation function for *mat A-3* in yeast. The *mat A-3* gene fused with *GAL4-BD* led to the growth of HF7c transformants in SD media devoid of tryptophan and histidine (Figure 1B). However, these transformants were unable to grow on the same medium enriched with 2.5 mM 3-AT (Figure 1C). The assay with the Y190 transformants carrying the *mat A-3* sequence fused to GAL4p-BD showed a weak β-galactosidase activity in the filter assay (Figure 2E), which supports the proposed transcriptional activity function for mat A-3.

Indication of transcriptional activation in the yeast reporter system was previously shown for *N. crassa* mat a-1, mat A-1 and mat A-3 proteins (Badgett and Staben, 1999). Using the yeast two-hybrid technique, Jacobsen *et al.* (2002) showed transcriptional activity of *S. macrospora* SMTA-1 and SMTa-1 mat proteins. However, the homologues of *N. crassa mat A-2* and *mat A-3* present in *S. macrospora* (*SmtA-2* and *SmtA-3,* respectively) did not show any yeast reporter gene activation. The *SmtA-3* gene has high similarity to the 5' end of the *N. crassa mat A-3* gene and its 3' end is more similar to the *N. crassa mat a* idiomorph and does not include the HMG domain (Pöggeler *et al.*, 1997). It is probably due to these differences that the two related proteins show a different behavior in the yeast reporter gene system. The homologue of *mat A-3* in *P. anserina (the SMR2* gene) encodes a protein which, along with FMR1, seems to function as a repressor of the *mat+* and as an activator of *mat-* specific functions (Arnaise *et al.*, 2001). Based on the sequence similarity of *N. crassa mat A-3* with *P. anserina SMR2* this hypothesis supports the potential gene activation observed for the mat A-3 protein (Ferreira *et al.*, 1996).

Since mat A-2 did not show any transcriptional activity and mat A-3 showed only a weak activation separately, it is possible that an interaction of the mat proteins is necessary for activation or repression of transcription, as suggested by *in vivo* analysis (Ferreira *et al.*, 1998). In *S. cerevisiae*, **a1** and α2 proteins heterodimerize in the diploid cells, turning off the expression of α*1*, which is a positive regulator of α-specific genes, and of several haploid-specific genes (reviewed in Souza *et al.*, 2003). Heterodimerization of mating-type proteins is also observed in Basidiomycete fungi and the regulatory heterodimer formed is responsible for intracellular recognition of sexual compatibility (reviewed in Bakkeren *et al.*, 2008). Initially, Glass and Staben (1990) postulated a model for *N. crassamat* genes function where, in the vegetative phase, they regulate A- and a-specific genes along with those involved with vegetative incompatibility. When fertilization occurs, the mat proteins would interact to form a regulatory activator of the perithecium-differentiation genes. Genetic and RNA analysis of mat mutants also indicate that mat proteins form a complex (Ferreira *et al.* 1998).

Plasmids pADA-2 (*GAL4-AD-mat A-2*) and pADA-3 (*GAL4-AD-mat A-3*) were constructed for the two-hybrid analysis. Both plasmids were transformed into yeast in different combinations with plasmids pBDA-2 (*GAL4-BD-mat A-2*) and pBDA-3 (*GAL4-BD-mat A-3*). This would allow identifying hetero or homodimerization of *N. crassa* mat A-2 and mat A-3 proteins. Since the *N. crassa* mat proteins were not supposed to bind to the *GAL1* promoter sequence, which is the promoter used for the reporter genes, HF7c and Y190 strains were transformed with pADA-2 or pADA-3 to serve as negative control. HF7c and Y190 transformants were then analyzed for expression of their respective reporter gene (Figures 3  and 4). In the X-gal assay, pBDA-3/pADA-2 (Figure 3F) showed a weak transcriptional activation of the *LacZ* reporter gene, indicating weak interaction between mat A-2 and mat A-3 proteins; pBDA-3/pADA-3 (Figure 3G) resulted in an ambiguous signal, suggesting that a weak mat A-3/mat A-3 interaction may have occurred. This transcriptional activity seemed to be stronger than that observed for the *mat A-3* sequence alone (see Figure 2E for comparison).

HF7c transformants carrying the combinations pBDA-3/pADA-3 and pBDA-3/ pADA-2 were able to grow on SD media lacking tryptophan and histidine and enriched with 2.5 mM 3-AT; some growth in the presence of 5 mM 3-AT was also observed (Figure 4B and 4). In addition to the suggestion of homodimerization of mat A-3 protein and heterodimerization of mat A-2 and mat A-3 proteins, the 3-AT two-hybrid test also indicated that both interactions are stronger than the transcriptional activation function of the mat A-3 protein alone (see Figure 1C for comparison).

Surprisingly, the pADA-3 transformant (*mat A-3* gene fused to *GAL4-AD*) showed a weak transcriptional activation in the filter assay (Figure 3C) although no growth of HF7c transformed with the same plasmid was observed in SD media lacking histidine (Figure 4A-2). This indicated that the *N. crassa* mat A-3 protein was also capable of binding to DNA, as expected for HMG proteins, but the interaction was not very strong. The HMG domain is a DNA-binding motif present in transcriptional regulator proteins involved in cell differentiation and non-histone elements of the chromatin, including the SRY-family; some members of the HMG family of proteins bind to DNA with low sequence specificity (Grosschedl *et al.*, 1994). Philley and Staben (1994) demonstrated that the HMG sequence of mat a-1 protein was capable of binding *in vitro* to the 5'-CTTTG-3' sequence. It was also reported that the mat A-3 protein could bind *in vitro* to the same sequence (Philley and Staben, unpublished data cited in Ferreira *et al.*, 1996). The 907 bp sequence of the *GAL1* promoter (GenBank K02115) contains a unique 5'-CTTTG-3' sequence at position 121 and its complement (5'-CAAAG-3') at position 186. These are not included in the *GAL1*_*UAS*_ sequence (298-663 bp region) (Guarente *et al.*, 1982; Yocum *et al.*, 1984; GenBank K02115) which is the promoter used for the HIS3 reporter in the HF7c and the lacZ reporter in Y190. However, there are at least three 5'-A/TAACAAT/A-3' sequences, that are known to be DNA binding motifs for HMG proteins in the *GAL1*_*UAS*_ sequence. It is possible that the HMG domain of the mat A-3 protein recognized one or more of these DNA-binding sites in the *GAL1*_*UAS*_ promoter with low sequence specificity.

Not even a low transcription level of the *HIS3* reporter gene was observed in HF7c cells transformed with pADA-3 (*GAL4p-mat A-3*). Since we are assuming that the mat A-3 protein interacted with low specificity with the DNA-binding sites present in the *GAL1* promoter, the absence of growth in the HF7c transformant probably occurred because the interaction was not strong enough to activate transcription to a level that could allow growth of the transformant in SD – his.

In our two-hybrid experiments, a weak interaction between mat A-2 and mat A-3 was observed when these proteins were fused to GAL4p-AD and GAL4p-BD domains, respectively. It may be that the proper folding of mat A-2 and/or mat A-3 was not established when the fusion was made with GAL4p-BD and GAL4p-AD, respectively. Our result, along with that of Badgett and Staben (1999), supports the hypothesis of mating-type proteins working in a complex to activate or repress genes in different developmental pathways (Ferreira *et al.*, 1998). Curiously, in *P. anserina* protein-protein interaction in two-hybrid assays was only seen between the FMR1 and SMR2 proteins (Debuchy and Coppin, unpublished data cited in Coppin *et al.*, 1997). It is known that all four *P. anserina mat* genes are necessary for the post-fertilization events (Debuchy *et al.*, 1993); and FPR1 and the heterodimer FMR1/SMR2 are responsible for internuclear recognition, while SMR1 protein is required for dikaryotic cell formation (Zickler *et al.*, 1995; Arnaise *et al.*, 1997). The mat A-2 and mat A-3 proteins are most likely both involved with multiple nuclei mitotic divisions before migration into the crozier, since the few progeny observed in crosses with the double mutants is totally biparental (Ferreira *et al.*, 1998). Thus, our results support the idea that *N. crassa* and *P. anserina* differ from one another in the control of post-fertilization events, as was postulated in previous studies (Ferreira *et al.*, 1996, 1998).

Expression of some proteins in yeast can cause a toxic phenotype which may be detected by growth retardation and even death (Nonaka *et al.*, 2000; Roopchand *et al.*, 2001; Blanco *et al.*, 2003; Boyer *et al.*, 2004; Buryskova *et al.*, 2004). During our experiments, pBDA-2 (*GAL4p-BD-mat A-2*) and pBDA-2/pADA-3 (*GAL4p-AD-mat A-3*) transformants grew slower than other transformants. In addition, a new transformation with the pBDA-2/pADA-3 into HF7c cells had to be performed three times for completing the experiments, since the transformants survived less than four weeks in solid media. This is a rather short time compared to the other transformants which were viable for over a month under the same conditions. Also, the transformation frequency was always lower with pBDA-2 (not shown). It was thus hypothesized that the pBDA-2 plasmid could be toxic to yeast cells. In an attempt to test this hypothesis, growth of these transformants was analyzed over time.

The lag phase of both pBDA-2 and pBDA-2/pADA-3 transformants was longer, taking at least two additional hours before they achieved the log phase (not shown). This was more noticeable in the HF7c background which normally grows slower than the Y190 strain (data not shown). Conversely, the pBDA-3 transformants showed the shortest lag phase. At this phase, the metabolism of yeast cells changes in order to maximally benefit from the new environment. This is manifest in the strongly enhanced expression of proteins involved in the metabolism of carbohydrate and amino acids as well as protein synthesis (Brejning *et al.*, 2003). It is possible that the presence of mat A-2 protein fused to the GAL4p-BD domain interferes with the adaptation of the yeast cells to the new environment. This could explain the increase in the lag phase observed in transformants carrying plasmid pBDA-2. The mat A-3 protein fused to the GAL4p-BD domain, conversely, could be helping the yeast cells in this adaptation, thus shortening the lag phase.

Although the lag phase was increased in all mat A-2 transformants, no significant difference was observed at the exponential phase, as indicated by the cell doubling time (p > 0.05). No difference in doubling time was detected between HF7c transformants bearing pBDA-2 and pBDA-2/pADA-3. However, the doubling time of these two transformants is longer than the one observed for the HF7c pGBT9 transformants (Table 2).

Being interested in the interference of *N. crassa* mating proteins on the growth of *S. cerevisiae*, we conducted a plasmid cure experiment. Y190 cells harboring the four different constructs were grown without selective pressure (YPD) and then plated on SD lacking the specific marker amino acids and on YPD plates as control (Table 2). Ninety to 100% of the plated pGBT9 transformants were able to grow on selective media. Transformants harboring mat A-3 also had a high survival rate (68%-72%). However, cells initially harboring the mat A-2 gene, either alone or in combination with mat A-3, showed a dramatic loss of the markers in the absence of selective pressure (4.8%-13.2%), indicating a toxic effect of this construct on yeast.

The *N. crassa* mating-type proteins that were expressed in *S. cerevisiae* in this study are thought to be developmentally regulated and only functional in the sexual cycle (Ferreira *et al.*, 1996). Our initial experiments were performed using only the mitotic cell cycle of this yeast, and thus both mat proteins were expressed during a cell cycle in which they normally would be silenced. Although mat A-2 and mat A-3 do not exhibit a domain similar to the MAT proteins of *S. cerevisiae* we wondered whether the mating-type and ploidy state of the yeast receiver would make any difference in the growth of the transformants. Plasmids pGBT9, pBDA-2, pDBA-3 were transformed into isogenic haploid (IH1783a and IH 1784α) and diploid cells (IH1788a/α). These transformants were grown on selective media and their growth rates were measured (Table 3). The ploidy apparently did not make any difference since the doubling time of the diploid transformant was not affected by the presence of the mat genes (p > 0.05). Apparently in the mat α background the doubling times for mat A-2 transformants were higher. However, statistical tests showed no significant difference probably because of the high variance between the repetitions (data not shown). Thus we cannot affirm that *N. crassa* proteins have an effect in the yeast cell cycle.

A curious aspect is that, although mat A-2 and mat A-3 proteins seem to have similar functions in *N. crassa* (Ferreira *et al.* 1998), they displayed different behavior in the two-hybrid experiment. Nevertheless, a more clear understanding about the roles of the mat A-2 and mat A-3 proteins can only be reached when their target genes are identified. Galgoczy *et al.* (2004) were able to identify a large number of specific target genes of *S. cerevisiae* mat proteins using genome-wide chromatin immunoprecipitation, transcriptional profiling and phylogenetic comparisons. Different studies aiming at analyzing mat-regulated genes in sexual and non-sexual fungi demonstrate the importance of understanding the control of vegetative and sexual cycles in filamentous fungi (Nowrousian *et al.*, 2005; Pögeller *et al.* 2006; Keszthelyi *et al.*, 2007). In this context, the pattern of interaction of mat proteins studied here contributes towards understanding the control of vegetative and sexual cycles in *N. crassa* and in other fungal species.

## Figures and Tables

**Table 1:** - Sequences of *mat A-2* and *mat A-3* primers used in this study.

Primer	Sequence (5' - 3')	Gene
EDGE-F	CAGAATTCGACATCAATCTTCTCAACATGCAA	*mat A-2*
EDGE-R	TTGCTGCAGCCACGGATTCTACCATCCCA	*mat A-2*
BONO-F	GTGAATTCCGTCTGCACCCCTCTATCACA	*mat A-3*
BONO-R2	GTGAATTCTTGGTTGTTGATCAACT	*mat A-3*

Restriction enzyme sites are underlined. GAATTC - *Eco*R I, CTGCAG - *Pst* I.

**Table 2:** - Plasmid cure in Y190 cells.

	Number of colonies^a^			
Media	pGBT9	pBDA-2	pBDA-3	pBDA-2/pADA-3
YPD	189-208	165-242	161-288	156-280
SD -aa	307**-188	8-32	116-195	2-13

^a^Number of colonies per plate in two different experiments. **High number of colonies in this control may be due to plating error. pGBT9 - vector, pBDA-2 - *GAL4-BD-mat A-2*, pBDA-3 - *GAL4-BD-mat A-3*, pADA-3 - *GAL4-AD-mat A-3*.

**Table 3:** - Doubling time of different yeast cells transformed with pGBT9, pBDA-2 and pBDA-3.

Strain	Cell doubling time (mean in minutes)			
	pGBT9	pBDA-2	pBDA-3	pBDA-2/pADA-3
HF7c	151	183	147	200
Y190	112.5	129	116	185
IH1783(a)	135	124	116	-
IH1784(α)	153	191	132	-
IH1788(a/α)	140	169	123	-

pGBT9 - vector, pBDA-2 - *GAL4-BD-mat A-2*, pBDA-3 - *GAL4-BD-mat A-3*, pADA-3 - *GAL4-AD-mat A-3*.
